# Draft Genome Sequences of Three Clinical Isolates of Madurella mycetomatis, the Major Cause of Black-Grain Mycetoma

**DOI:** 10.1128/MRA.01533-19

**Published:** 2020-04-16

**Authors:** El Shiekh Khidir, Abdalla Ahmed, Ahmed Hassan Fahal, Al Amin Ibrahim

**Affiliations:** a Laboratory Medicine Department, Faculty of Applied Medical Sciences, Umm Al-Qura University, Mecca, Saudi Arabia; b Department of Microbiology, Faculty of Medicine, Umm Al-Qura University, Mecca, Saudi Arabia; c Mycetoma Research Center, University of Khartoum, Khartoum, Sudan; d Department of Microbiology, Faculty of Medical Laboratory Sciences, Khartoum University, Khartoum, Sudan; University of California, Riverside

## Abstract

The draft genomes of three fungal clinical isolates of Madurella mycetomatis from patients with mycetoma are presented. No finished genome is currently available for this important fungus. Therefore, the addition of these new draft genomes will help us better understand the diversity and pathogenicity of this important species.

## ANNOUNCEMENT

Madurella mycetomatis is an ascomycetous dematiaceous fungus responsible for most black-grain eumycetoma worldwide (1, 2). This filamentous fungus produces few spores or no asexual spores, and therefore the mycelial growth is usually referred to as sterile mycelium (3). This sparse sporulation makes morphological identification difficult, requiring a better understanding of diagnostic markers at the genome level. *M. mycetomatis* is not the only species that causes black-grain mycetoma. Many other fungi belonging to different taxonomic orders are also known to produce black-grain mycetoma. However, *M. mycetomatis* is the main agent of eumycetoma, which is associated with increased morbidity, disability, and poor response to medical treatment, with profound socioeconomic consequences (4, 5). To date, only one draft genome of *M. mycetomatis* has been reported. Based on small subunit and internal transcribed spacer ribosomal DNA (rDNA) sequencing data, *M. mycetomatis* is now well classified in the class Ascomycetes, order Sordariales, with its closest species Chaetomium thermophilum (1).

In this study, *M. mycetomatis* clinical isolates were obtained from patients attending the mycetoma clinic at the Mycetoma Research Centre of Khartoum University (Khartoum, Sudan). Primary isolation of *M. mycetomatis* was performed by culturing washed black grains on Sabouraud’s dextrose agar with chloramphenicol to inhibit bacterial growth, followed by incubation at 37°C for 1 week. Subsequent genomic work was done at the Department of Microbiology at the College of Medicine, Umm Al-Qura University, in Saudi Arabia. *M. mycetomatis* strains were isolated by direct culture of the black grains obtained by a deep surgical biopsy and identified by morphology and PCR. Prior to DNA extraction, clinical isolates of *M. mycetomatis* were grown on Sabouraud’s dextrose agar plates, incubated at 37°C for 5 to 7 days. The DNA was extracted using phenol-chloroform (1:24, pH 8.0) and then precipitated using iso-propanol, washed with 70% ethanol, dried at room temperature, and resuspended in 35 μl Tris-EDTA (TE) buffer (pH 8.0). The DNA quantity and quality were determined using a Qubit fluorometer (Invitrogen, Applied Biosystems, USA) and an Agilent BioAnalyzer 2100 (USA) with a DNA 1000 chip (Agilent). Libraries were prepared using the Illumina Nextera XT DNA library preparation kit following the manufacturer’s protocol. Whole-genome sequencing was performed on a MiSeq platform using the 600-cycle sequencing kit v3 (Illumina, CA, USA). Paired-end sequence reads were retrieved from the Illumina MiSeq instrument and checked for quality with the FastQC tool (6). The overall quality of sequence reads was acceptable, and during assembly with DNASTAR, all low-quality and short reads were trimmed. Sequencing the three isolates resulted in 5,160,000 reads for strain NMM13, 5,480,000 reads for strain NMM18, and 7,020,000 reads for strain NMM15. The average GC content was 53%. *De novo* genome assembly was performed with SeqMan NGen v14.1.0 (DNASTAR, Madison, USA) with default settings, resulting in 3,990 contigs for strain NMM13, 16,963 contigs for strain NMM15, and 5,814 contigs for strain NMM18. The average sequencing depth was 34×, and the average sequence quality of the assembled sequences was 34 (quality measured by Phred score). The assembly resulted in contigs with *N*_50_ values of 15 kb for strain NMM13, 10 kb for strain NMM18, and 2 kb for strain NMM15.

### Data availability.

The sequence reads of the draft genomes of the three clinical *M. mycetomatis* strains have been deposited in the NCBI Sequence Read Archive under accession number PRJNA579746. Table 1 lists the genome data and accession numbers for the three strains.

**TABLE 1:** Genome data and accession numbers of three *M. mycetomatis* clinical isolates

Strain	No. of contigs	Genome coverage (×)	Total no. of reads	Avg contig length (bp)	Contig *N*_50_ (kb)	GC content (%)	GenBank accession no.	SRA accession no.
NMM13	3,990	39	5,160,000	93,073	15	53.67	WOGW00000000	SRS5801849
NMM15	16,963	26	7,020,000	16,300	2	53.71	WOGY00000000	SRS5801850
NMM18	5,814	39	5,480,000	59,340	10	53.25	WOGX00000000	SRS5801851

## References

[1] Nenoff P, van de Sande WWJ, Fahal AH, Reinel D, Schöfer H 2015 Eumycetoma and actinomycetoma—an update on causative agents, epidemiology, pathogenesis, diagnostics and therapy. J Eur Acad Dermatol Venereol 29:1873–1883. doi:10.1111/jdv.13008. 25726758

[2] van de Sande WWJ 2013 Global burden of human mycetoma: a systematic review and meta-analysis. PLoS Negl Trop Dis 7:e2550. doi:10.1371/journal.pntd.0002550. 24244780 PMC3820768

[3] Ahmed SA, van de Sande WWJ, Desnos-Ollivier M, Fahal AH, Mhmoud NA, de Hoog GS 2015 Application of isothermal amplification techniques for identification of Madurella mycetomatis, the prevalent agent of human mycetoma. J Clin Microbiol 53:3280–3285. doi:10.1128/JCM.01544-15. 26246484 PMC4572522

[4] Sharma SK, Mukherjee A, Singh AK, Seth T, Kumar S, Mishra P, Xess I, Gupta S, Mahapatra M, Pati H 2012 Madurella mycetomatis infection following allogenic stem cell transplantation for aplastic anemia. Mediterr J Hematol Infect Dis 4:e2012038. doi:10.4084/mjhid.2012.038. 22811787 PMC3395676

[5] Estrada-Chavez GE, Vega-Memije ME, Arenas R, Chavez-Lopez G, Estrada-Castañon R, Fernandez R, Hay R, Dominguez-Cherit J 2009 Eumycotic mycetoma caused by Madurella mycetomatis successfully treated with antifungals, surgery, and topical negative pressure therapy. Int J Dermatol 48:401–403. doi:10.1111/j.1365-4632.2009.03967.x. 19335427

[6] Wingett SW, Andrews S 2018 FastQ Screen: a tool for multi-genome mapping and quality control. F1000Res 7:1338. doi:10.12688/f1000research.15931.1. 30254741 PMC6124377

